# Serum Proteomics Reveals Systemic Responses in *Didelphis aurita* Naturally Infected with *Hepatozoon* sp.

**DOI:** 10.3390/pathogens14101042

**Published:** 2025-10-14

**Authors:** Andrés Mauricio Ortega Orozco, Camilo Jose Ramirez-Lopez, Lucas Drumond Bento, Pollyanna Cordeiro Souto, Fabrícia Modolo Girardi, Veronica Rodrigues Castro, Edvaldo Barros, Joao Vitor Gonçalves de Oliveira, Renner Philipe Rodrigues Carvalho, Artur Kanadani Campos, Leandro Abreu da Fonseca

**Affiliations:** 1 Graduate Program in Veterinary Medicine, Federal University of Viçosa, Viçosa 36570-900, Brazil; amauricioortega@gmail.com (A.M.O.O.); camilo.lopez@ufv.br (C.J.R.-L.); lucasdrumond29@gmail.com (L.D.B.); pollyannasouto@hotmail.com (P.C.S.); fabricia.girardi@ufv.br (F.M.G.); veronica.r.castro@ufv.br (V.R.C.); joao.goncalves.oliveira@ufv.br (J.V.G.d.O.); renner.carvalho@ufv.br (R.P.R.C.); artur.kanadani@ufv.br (A.K.C.); 2 Biomolecule Analysis Center, Federal University of Viçosa, Viçosa 36570-900, Brazil; edvaldo.barros@ufv.br

**Keywords:** ecoimmunology marsupial biology, hemoparasite, mass spectrometry

## Abstract

*Didelphis aurita* is a widely distributed neotropical marsupial frequently found in peri-urban environments and known to harbor various pathogens, including hemoparasites of the genus Hepatozoon. However, the systemic physiological responses of naturally infected individuals remain poorly understood. This study aimed to characterize the serum proteomic profile of *Didelphis aurita* naturally infected with *Hepatozoon* sp., providing insights into host–parasite interactions and potential biomarkers of infection. Serum samples were analyzed using liquid chromatography–tandem mass spectrometry (LC-MS/MS), followed by functional annotation based on Gene Ontology and KEGG pathway enrichment. A total of 67 proteins were identified, 33 of which were exclusive to infected animals. The most abundant proteins included albumin, hemoglobin subunits, and venom metalloproteinase inhibitors (DM43 and DM64). Functional enrichment revealed significant involvement in complement and coagulation cascades, protease inhibition, antioxidant defense, and extracellular vesicle localization. Key proteins such as fibrinogen, plasminogen, antithrombin, SERPIN family members, vitronectin, and fibronectin suggest an integrated host response involving hemostasis, inflammation control, and tissue remodeling. This is the first report of the serum proteome of *Didelphis aurita* naturally infected with *Hepatozoon* sp. Despite the absence of protein validation, the findings provide novel insights into marsupial immunophysiology and offer a foundation for future biomarker research and ecoimmunological surveillance in synanthropic species.

## 1. Introduction

Opossums of the genus *Didelphis* are marsupials widely distributed across the American continent, ranging from temperate regions of North America to the subtropical areas of Patagonia. In Brazil, representatives of this genus are present throughout most of the national territory, with records from Paraíba in the northeast to Rio Grande do Sul in the far south [1]. Among these native species, the black-eared opossum (*Didelphis aurita*) is notable for its small size (up to 3 kg), predominantly crepuscular and nocturnal habits, omnivorous diet with insectivorous tendencies, and broad ecological adaptability [2,3]. In addition to its ecological roles as an insect predator and seed disperser, *D. aurita* possesses distinctive physiological and immunological traits, including a highly responsive immune system, which have supported its use as an experimental model in biomedical research, particularly in studies of infection and immune responses [4,5,6]. Investigating its interaction with parasites and arthropod vectors of medical and veterinary importance therefore represents an emerging field of ecoimmunological relevance.

Previous studies have demonstrated that Brazilian opossums can act as natural or experimental hosts for tick-borne pathogens, some of which have recognized zoonotic potential, positioning these animals as relevant targets for public health surveillance [7,8,9]. The tick *Ixodes loricatus*, frequently found parasitizing *D. aurita*, has been implicated as a vector of infectious agents in wildlife [10]. Among these pathogens, haemogregarines of the genus *Hepatozoon* (Apicomplexa: Adeleorina) deserve special attention. Depending on the host species, *Hepatozoon* infections may range from subclinical to severe, leading to anemia, splenomegaly, and immune dysfunction [11,12]. Although these protozoans have been reported in a wide array of vertebrates—including reptiles, carnivores, and rodents—information on marsupials is scarce, with only isolated records in *Didelphis albiventris* from Mato Grosso do Sul and São Paulo [13,14,15].

*Hepatozoon* spp. display a heteroxenous life cycle in which ixodid ticks serve as definitive hosts and vertebrates act as intermediate or paratenic hosts. Transmission to vertebrates occurs predominantly through ingestion of infected ticks or paratenic prey; after invasion, sporozoites disseminate to tissues (liver, spleen, and muscle) where merogony occurs, while circulating gamonts develop within leukocytes/erythrocytes. Sexual reproduction and sporogony take place in the tick midgut following a blood meal on an infected vertebrate. Clinically, hepatozoonosis spans a continuum from subclinical carriage to chronic disease marked by anemia, weight loss, splenomegaly, thrombocytopenia, and thrombo-inflammatory sequelae. Disease severity is influenced by parasite lineage/species, host species/age/immune status, parasitemia intensity, and co-infections with other tick-borne agents, and lineage-specific differences in virulence are well documented in carnivores and reptiles, with likely parallels in neotropical marsupials that remain unexplored. Within this eco-epidemiological context, serum proteomics provides a sensitive window into host processes—complement/coagulation, protease inhibition, extracellular vesicle signaling, and redox balance—expected to be engaged during hepatozoonosis, thereby reinforcing the rationale for applying this approach in *D. aurita*.

This paucity of data in marsupials contrasts with advances in other host species. In dogs and reptiles, proteomic and molecular studies have revealed systemic host responses and candidate biomarkers of *Hepatozoon* infection, highlighting the potential of such approaches for elucidating host–parasite interactions [11,12]. However, no equivalent investigations have been conducted in neotropical marsupials. Considering the ecological ubiquity of *Didelphis* spp., their synanthropic behavior, and their close interactions with domestic animals and humans, this gap limits evaluation of their potential role in hemoparasite transmission at the wildlife–domestic–human interface.

Recently, our research group identified *Hepatozoon* sp. in *D. aurita* individuals using both morphological (blood smears) and molecular (PCR) techniques, expanding the current knowledge on hemoparasite infections in neotropical marsupials [16]. Nonetheless, little is known about the physiological impacts of these infections on marsupials, despite their widespread distribution and frequent occurrence in peri-urban environments. Because *D. aurita* interacts closely with domestic animals and inhabits areas of human–wildlife interface, clarifying its role in hemoparasite transmission is important not only for marsupial biology but also for assessing potential zoonotic risks. In this context, proteomic approaches provide a powerful means of exploring systemic host responses and identifying biomarkers, yet no serum proteomic studies have been performed in *D. aurita* to date.

This study therefore aimed to characterize the serum proteomic profile of *D. aurita* naturally infected with *Hepatozoon* sp., providing novel insights into host–parasite interactions and supporting ecoimmunological surveillance.

## 2. Materials and Methods

### 2.1. Ethical Considerations

This study builds upon previous findings from our group on the detection of *Hepatozoon* sp. and other hemoparasites in *Didelphis aurita* [16] and aims to advance the understanding of host physiological responses through proteomic analysis. It was approved by the Ethics Committee on Animal Use of the Federal University of Viçosa (CEUA/UFV) under protocol number 30/2021. Authorization was also granted by the Brazilian Biodiversity Information and Authorization System (SISBIO), of the Brazilian Institute of Environment and Renewable Natural Resources (IBAMA), under license number 64930-3. All animals were released at the site of capture immediately after sampling, in accordance with ethical and legal requirements.

### 2.2. Study Area

The animals used in this study were collected in the municipality of Viçosa, in the state of Minas Gerais, Brazil (22°45′14″ S; 42°52′55″ W). The municipality covers an area of 299.418 km^2^ and has a humid subtropical climate, classified as Cwa according to the Köppen–Geiger system, with an average annual temperature of 20.4 °C and an average annual precipitation of 1251 mm (Available online: https://en.climate-data.org/south-america/brazil/minas-gerais/vicosa-25021/) (accessed on 15 February 2020).

### 2.3. Animal Capture, Sample Collection, and Experimental Grouping

Between March 2020 and July 2021, black-eared opossums (*Didelphis aurita*) were captured within the campus of the Federal University of Viçosa (UFV), located in Viçosa, Minas Gerais, Brazil, using baited Tomahawk live traps (0.45 × 0.21 × 0.21 m). Following capture and restraint, blood samples were collected via venipuncture of the lateral coccygeal vein, as described by Carvalho do Nascimento and Horta (2014) [2]. Samples were stored in EDTA tubes (Becton & Dickinson Co., Franklin Lakes, NJ, USA) for hematological analyses and in clot activator tubes (Becton & Dickinson Co., Franklin Lakes, NJ, USA) for biochemical analyses.

Parasitological screening was performed through the examination of Giemsa-stained blood smears to detect the presence of hemoparasites (Supplementary Figure S1). Based on smear results, animals were allocated into two experimental groups: the Hept-Positive group, comprising nine individuals in which *Hepatozoon* sp. gamonts were identified; and the Hept-Negative group, consisting of ten individuals in which no structures compatible with Hepatozoon or other hemoparasites were observed. Although experimental grouping was based on blood smear results, the presence of *Hepatozoon* sp. in Hept-Positive animals was previously confirmed by PCR, as detailed in Orozco et al. (2022) [16], thereby validating the infection status of both groups.

### 2.4. Protein Extraction and Quantification

Protein extraction was performed using 100 µL of serum, which was precipitated with 600 µL of a chilled solution containing acetone, trichloroacetic acid (10% *w*/*v*), and dithiothreitol (DTT, 1 mM). The resulting protein pellet was resuspended in 250 µL of a solubilization buffer composed of 7 M urea, 2 M thiourea, 4% CHAPS (3-[(3-cholamidopropyl) dimethylammonio]-1-propanesulfonate), and 40 mM DTT.

Protein quantification was performed based on a standard calibration curve prepared with known concentrations of bovine serum albumin (BSA). The BSA standards were mixed with 150 µL of Bradford reagent (1×), and ultrapure water was added to reach a final volume of 200 µL. After incubation at room temperature for 10 min, absorbance readings were taken at 595 nm using a spectrophotometer (Multiskan SkyHigth, Thermo Scientific, Waltham, MA, USA). Triplicates containing only ultrapure water were used as blanks. The average absorbance of each sample was corrected by subtracting the mean values of the blanks and empty wells, and a linear regression was fitted to generate the standard curve. Serum protein concentrations were then determined based on this calibration curve, following the method described by Bradford [17].

### 2.5. Protein Processing by Electrophoresis

To enhance proteomic coverage and mitigate individual variability, serum samples were pooled within each group (Hept-Positive and Hept-Negative) prior to SDS-PAGE and LC-MS/MS. Pooling was justified by the exploratory nature of this study, the limited sample volumes obtained from wild animals, and the need to ensure sufficient protein yield for high-quality LC-MS/MS analysis. This strategy provided a representative overview of group-level proteomic patterns but limited the ability to assess inter-individual variation or perform inferential statistics. Accordingly, results should be interpreted as exploratory and descriptive.

Total protein extracts were first evaluated individually by one-dimensional sodium dodecyl sulfate–polyacrylamide gel electrophoresis (SDS-PAGE), as described by Laemmli [18], to verify the quality of protein extraction and quantification (Supplementary Figure S2). Subsequently, 60 µg of protein from each sample were pooled to generate representative samples of each experimental group. For each group, five pooled replicates containing 50 µg of protein were processed using the SDS-PAGE short-run strategy, employing a 4% stacking gel and a 12.5% resolving gel [19,20]. Electrophoresis was performed at a constant voltage of 80 V and halted when the bromophenol blue tracking dye reached ~10 mm into the resolving gel. This short-run approach was chosen to concentrate the proteome in a compact band, facilitating efficient in-gel digestion while reducing contaminating background proteins [21].

### 2.6. Enzymatic Digestion and Desalting

Protein bands obtained from SDS-PAGE short-run were excised, finely chopped with a sterile blade, and subjected to enzymatic digestion following the protocol described by Shevchenko et al. (2006) [22]. Briefly, gel fragments were placed in 1.5 mL microtubes containing 200 µL of 50% (*v*/*v*) acetonitrile in 25 mM ammonium bicarbonate (pH 8.0) and washed to remove Coomassie stain and residual SDS. Subsequently, gels were dehydrated twice with 200 µL of 100% acetonitrile, with 5 min incubations at room temperature. The fragments were then dried for 15 min using a vacuum centrifuge (model AG-22331, Eppendorf, Hamburg, Germany).

Protein reduction was performed using 100 µL of 65 mM DTT in 100 mM ammonium bicarbonate (pH 8.0) at 56 °C for 30 min. Alkylation was carried out with 100 µL of 200 mM iodoacetamide in 100 mM ammonium bicarbonate (pH 8.0), incubated at room temperature for 30 min in the dark. The gel pieces were then washed twice with 100 mM ammonium bicarbonate followed by dehydration with acetonitrile and finally dried again in a vacuum centrifuge.

For proteolysis, the gel fragments were rehydrated on ice with 100 µL of TPCK-treated porcine trypsin (Promega, V5111, Madison, WI, USA), at a final concentration of 25 ng/µL in activation buffer (40 mM ammonium bicarbonate, pH 8.0, and 10% acetonitrile). After 45 min on ice, 130 µL of the same activation buffer were added to each tube. Samples were incubated in a water bath at 37 °C for 22 h.

Following digestion, samples were sonicated for 10 min and vortexed for 20 s. The supernatant was transferred to clean microtubes. Then, 150 µL of a solution containing 5% (*v*/*v*) formic acid in 50% (*v*/*v*) acetonitrile were added to the remaining gel pieces. Tubes were vortexed for 20 s, left at room temperature for 15 min, and sonicated for an additional 2 min. The solution was combined with the previously collected digest. This extraction step was repeated once more, and all collected fractions were pooled and concentrated using a vacuum centrifuge.

Tryptic peptide desalting was performed using reversed-phase C18 microcolumns (model ZTC18S096, Millipore, Bedford, MA, USA), hereafter referred to as the stationary phase. The procedure followed the manufacturer’s instructions. Peptides were resuspended in 10 µL of 0.1% trifluoroacetic acid (TFA). The column was activated with 100% acetonitrile, equilibrated with 0.1% TFA, and the sample was loaded in 0.1% TFA solution. Desalting was achieved by washing with pure 0.1% TFA, and peptides were eluted with 100% acetonitrile. The final elution was combined with the column wash. The total recovered peptide solution was concentrated using a vacuum centrifuge. Dried samples were stored at −20 °C until mass spectrometry analysis.

### 2.7. Mass Spectrometry

Peptides obtained from the SDS-PAGE short-run replicates were solubilized in 20 µL of an aqueous solution containing 0.1% formic acid and 2% (*v*/*v*) LC-MS grade acetonitrile. The samples were transferred to appropriate vials for nano LC-MS/MS analysis. For each run, 1 µL of the peptide solution was injected into an ultra-high-performance liquid chromatography system (UHPLC NanoAcquity, Waters, Milford, MA, USA), equipped with a trap column (nanoAcquity UPLC^®^ 2G-V/MTrap 5 µm Symmetry^®^ C18, 180 µm × 20 mm, Waters), operating at a flow rate of 7 µL/min for 3 min.

Peptide separation was carried out on a nanoAcquity UPLC^®^ 1.8 µm HSS T3 analytical column (75 µm × 200 mm, Waters), at a flow rate of 0.2 µL/min. The mobile phase consisted of two solvents: water with 0.1% formic acid (solvent A) and acetonitrile with 0.1% formic acid (solvent B). The chromatographic gradient was programmed as follows: 2% B for 1 min; linear increase from 2% to 30% B over 209 min; increase from 30% to 85% B in 10 min; hold at 85% B for 5 min; decrease from 85% to 2% B in 5 min; and equilibration at 2% B for 10 min, totaling 240 min of run time.

Eluted peptides were automatically introduced into a MAXIS 3G mass spectrometer (Bruker Daltonics, Bremen, Germany) operating online with a CaptiveSpray ionization source. Proteomic analyses were conducted using an optimized method (IE_captive_nov2019), with a drying gas flow of 3 L/min, ion source temperature of 150 °C, and a transmission voltage of 2 kV. Raw data files were converted to *.mzXML format (Extensible Markup Language) using CompassXport software, version 3.0 (Bruker Daltonics), and peptide identification was performed using PEAKS software (Bioinformatics Solutions Inc., Waterloo, ON, Canada) [23].

### 2.8. Mass Spectrometry Data Analysis

The mass lists in mzXML format were searched against the Didelphidae protein database (downloaded on 23 August 2021; 39,466 entries), as deposited in the UniProt Consortium. The searches were conducted using PEAKS software, version 7.0 (Bioinformatics Solutions Inc., Waterloo, ON, Canada) [23].

The following search parameters were applied: trypsin enzymatic digestion with no missed cleavages allowed; carbamidomethylation of cysteine as a fixed modification; and methionine oxidation as a variable modification. Mass tolerance was set to 20 ppm for precursor ions and 0.6 Da for fragment ions, considering charge states of +2, +3, and +4. Proteins were considered confidently identified when at least two unique peptides were detected with a false discovery rate (FDR) < 1%. Proteins annotated as “Uncharacterized” were further analyzed using the BLAST tool (version 2.4.0) (Altschul et al., 1990) [24], to identify the most similar sequences in the non-redundant (nr) NCBI protein database.

Physicochemical properties such as molecular weight, isoelectric point, amino acid composition, and other parameters were calculated using the ProtParam tool (https://www.web.expasy.org/protparam/) (accessed on 25 August 2021), available through the Swiss Bioinformatics Resource Portal—Expasy [25].

Proteins were classified as “exclusive” if they were detected in one group (Pos-D or Pos-E) with at least two unique peptides and were completely absent from the other group. No fold-change thresholds were applied because pooling precluded quantitative abundance testing. Relative protein abundance was estimated by calculating the ratio between the sum of MS2 spectra assigned to the peptides of a given protein and the total number of MS2 spectra detected in the sample, with results expressed as percentages [26].

### 2.9. Functional Classification of Proteins

All identified proteins were functionally classified using the Database for Annotation, Visualization and Integrated Discovery (DAVID) (https://davidbioinformatics.nih.gov/tools.jsp) (accessed on 26 August 2021), based on functional categories, Gene Ontology (GO) annotations, and Kyoto Encyclopedia of Genes and Genomes (KEGG) metabolic pathways [27]. Pathway enrichment significance was evaluated using FDR-adjusted *p*-values, with thresholds set at *p* < 0.05.

## 3. Results

### 3.1. Proteomic Profile Characterization

**Figure 1:**
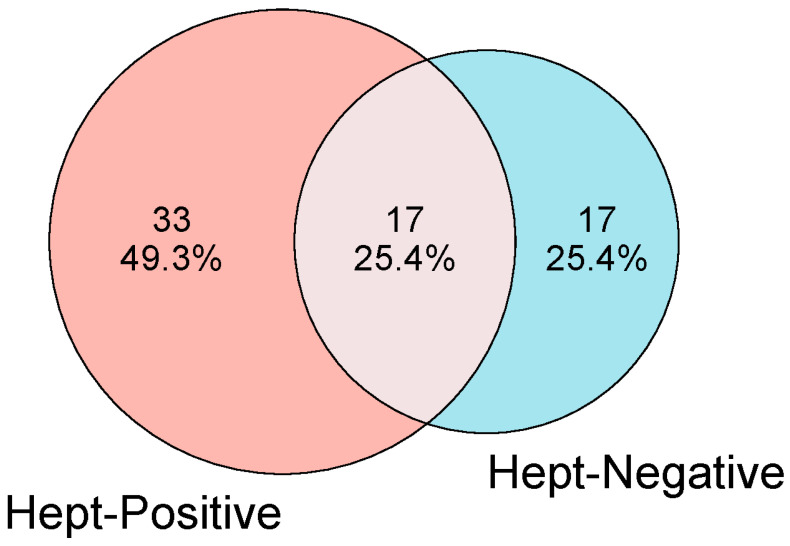
Venn diagram showing the distribution of serum proteins identified by LC-MS/MS across the experimental groups: *Hepatozoon-positive* animals (Hept-Positive) and *Hepatozoon-negative* animals (Hept-Negative). Numbers within the diagram indicate proteins unique to each group or shared between them, with percentages calculated relative to the total proteins identified.

Proteomic analysis by LC-MS/MS enabled the identification of 67 proteins in the serum of *Didelphis aurita*. Proteins were classified as exclusive when detected in one group with at least two unique peptides and completely absent from the other group, as detailed in Methods. Because samples were pooled, analyses are based on qualitative presence/absence rather than quantitative differential expression. Among the identified proteins, 33 were exclusively found in the Hepatozoon-positive group (Hept-Positive), based on the visualization of gamonts in blood smears. In contrast, 17 proteins were exclusively detected in the Hepatozoon-negative group (Hept-Negative), in which no hemoparasites were identified by this technique. The remaining 17 proteins were common to both groups (Figure 1). This qualitative distribution highlights that infection with Hepatozoon is associated with a broader diversity of serum proteins (33 exclusive to Hept-Positive vs. 17 to Hept-Negative).

Among the identified proteins, those with the highest relative abundance were albumin (ALB) (13%), hemoglobin subunit beta-M (10%), hemoglobin subunit alpha (8%), venom metalloproteinase inhibitor DM43 (7%), and venom myotoxin inhibitor DM64 (7%) (Table 1). These high-abundance proteins highlight fundamental systemic functions: ALB contributes to osmotic balance and transport of metabolites; hemoglobin subunits reflect oxygen transport and potential oxidative stress dynamics; while DM43 and DM64, known inhibitors of snake venom toxins, point to adaptive immunophysiological mechanisms in marsupials that may influence host–parasite interactions.

### 3.2. Functional Classification of Identified Proteins

**Figure 2:**
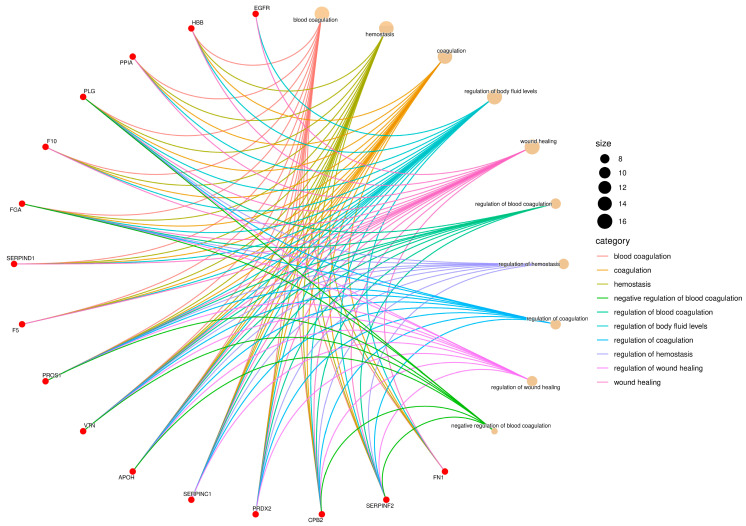
Gene Ontology (GO) enrichment network of the Biological Process (BP) category. Red nodes represent proteins identified in *Didelphis aurita* serum, and beige nodes represent enriched GO terms. Node size is proportional to the number of proteins annotated to each term, and edges are color-coded by GO category. Key processes included blood coagulation (12 proteins, FDR-adjusted *p* < 0.05), hemostasis (10 proteins, FDR-adjusted *p* < 0.05), and wound healing (8 proteins, FDR-adjusted *p* < 0.05).

**Figure 3:**
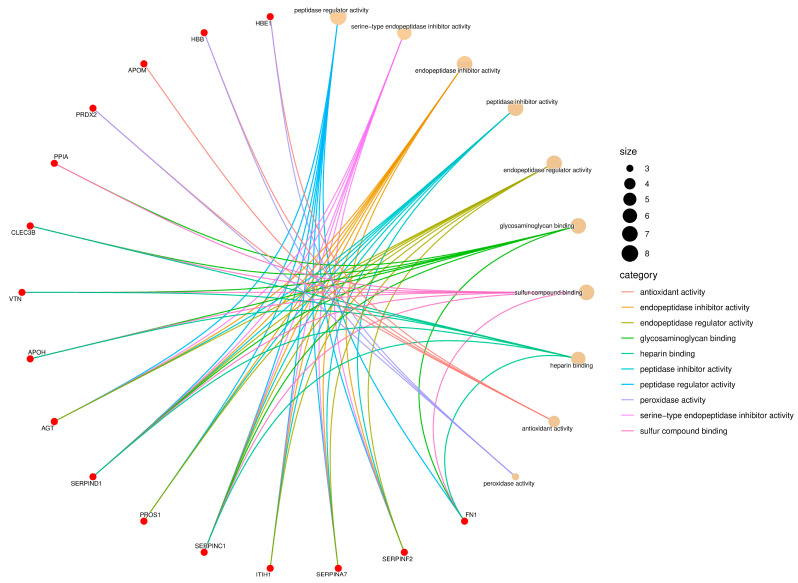
Gene Ontology (GO) enrichment network of the Molecular Function (MF) category. Red nodes represent proteins identified in *Didelphis aurita* serum, and beige nodes correspond to enriched GO terms. Node size reflects the number of proteins associated with each function, and edges are color-coded by GO category. Major functions included serine-type endopeptidase inhibitor activity (8 proteins, FDR-adjusted *p* < 0.05), glycosaminoglycan binding (6 proteins, FDR-adjusted *p* < 0.05), antioxidant activity (5 proteins, FDR-adjusted *p* < 0.05), and peroxidase activity (4 proteins, FDR-adjusted *p* < 0.05).

**Figure 4:**
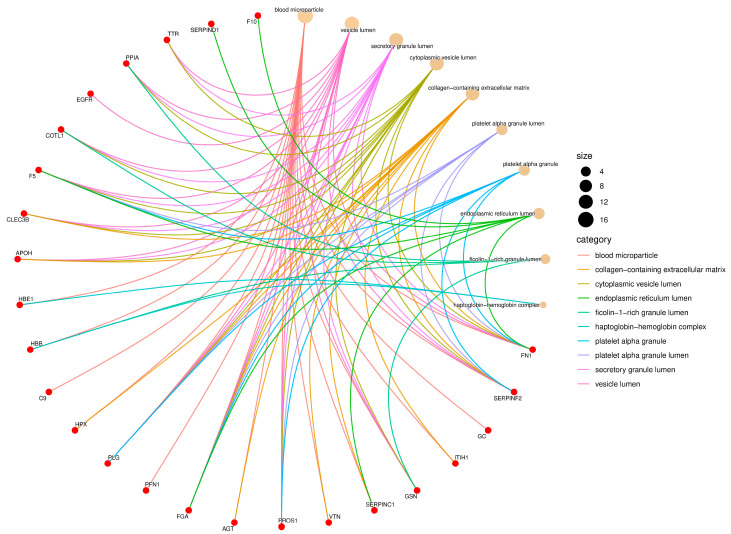
Gene Ontology (GO) enrichment network of the Cellular Component (CC) category. Red nodes represent proteins identified in *Didelphis aurita* serum, and beige nodes represent enriched cellular compartments. Node size is proportional to the number of proteins mapped to each compartment, and edges are color-coded by GO category. Enriched components included vesicle lumen (11 proteins, FDR-adjusted *p* < 0.05), platelet alpha granule lumen (9 proteins, FDR-adjusted *p* < 0.05), extracellular matrix (7 proteins, FDR-adjusted *p* < 0.05), and blood microparticles (7 proteins, FDR-adjusted *p* < 0.05).

The proteins identified in the serum of *Didelphis aurita* were functionally classified according to Gene Ontology (GO), encompassing the categories of Biological Process, Molecular Function, and Cellular Component. Enrichment analysis revealed a predominance of functions related to hemostasis, protease regulation, molecular binding, antioxidant activity, and localization in extracellular vesicles and platelet granules, as illustrated in Figure 2, Figure 3 and Figure 4.

In the Biological Process category, there was strong enrichment in terms such as blood coagulation, hemostasis, regulation of coagulation, wound healing, and regulation of body fluid levels (Figure 2). These terms involved 12 proteins with FDR-adjusted *p* < 0.05, including fibrinogen (FGA), coagulation factor V (F5), coagulation factor X (F10), plasminogen (PLG), VTN, antithrombin (SERPIND1), alpha-1-antiproteinase (SERPINA1), serpin family F member 2 (SERPINF2), carboxypeptidase B2 (CPB2), fibronectin (FN1), apolipoprotein H (APOH), and hemoglobin subunit beta-M (HBB). The terms regulation of blood coagulation and regulation of hemostasis were among the most connected, suggesting a central role of these pathways in the host response to parasitic infection.

For Molecular Function, the enriched terms included serine-type endopeptidase inhibitor activity, peptidase regulator activity, glycosaminoglycan binding, heparin binding, sulfur compound binding, and peroxidase activity (Figure 3). Proteins such as SERPINA7, SERPIND1, SERPINF2, inter-alpha-trypsin inhibitor heavy chain H3 (ITIH1), vitamin K-dependent protein (PROS1), FN1, APOH, VTN, and glutathione peroxidase (GPx) were involved in multiple of these functions. The high representation of protease inhibitors suggests an active modulation of proteolytic cascades, while the presence of antioxidant proteins like GPx and HBB indicates a potential defense against oxidative stress triggered by infection, which aligns with the enrichment of pathways associated with redox and metabolic regulation observed in KEGG.

In the Cellular Component category, most identified proteins were associated with extracellular compartments, notably vesicle lumen, secretory granule lumen, platelet alpha granule, collagen-containing extracellular matrix, blood microparticles, and the haptoglobin–hemoglobin complex (Figure 4). Proteins such as FN1, FGA, PLG, hemopexin (HPX), complement component C9, HBB, gelsolin (GSN), and various SERPIN family members were mapped to multiple compartments, reflecting their wide distribution and functional versatility in serum. The strong enrichment of terms related to platelet granules further emphasizes the relevance of platelet-derived and secretory proteins in the circulating proteome of *Didelphis aurita*.

Importantly, the enrichment of coagulation- and protease-related functions observed in the GO analysis was consistent with KEGG pathway enrichment results, where complement and coagulation cascades were also the most significantly represented.

### 3.3. Enrichment of Metabolic Pathways

**Figure 5:**
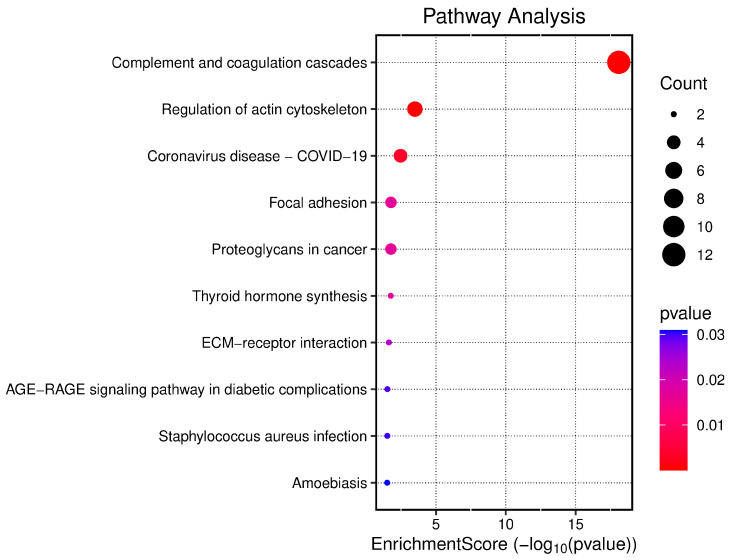
Dot plot of KEGG pathway enrichment analysis based on the proteins identified in the serum of *Didelphis aurita*. The size of each dot indicates the number of proteins associated with the corresponding pathway, while the color gradient represents the statistical significance of enrichment (FDR-adjusted *p*, –log_10_ scale). The Complement and coagulation cascades pathway showed the highest enrichment (13 proteins, FDR-adjusted *p* < 0.01), followed by Regulation of actin cytoskeleton (5 proteins, FDR-adjusted *p* < 0.05), Focal adhesion (4 proteins, FDR-adjusted *p* < 0.05), and ECM–receptor interaction (3 proteins, FDR-adjusted *p* < 0.05). The appearance of the “Coronavirus disease—COVID-19” pathway (3 proteins, FDR-adjusted *p* < 0.05) should be interpreted with caution, as it reflects proteins involved in broader inflammatory and immune processes rather than a direct association with viral infection.

Pathway enrichment analysis based on the KEGG database revealed the involvement of *Didelphis aurita* serum proteins in several biologically relevant processes (Figure 5). The most significantly enriched pathway was Complement and coagulation cascades, both in terms of the number of associated proteins and statistical significance (13 proteins, FDR-adjusted *p* < 0.05), highlighting the central role of these proteins in systemic hemostatic and inflammatory responses.

Additional pathways related to cell dynamics and immune regulation were also prominent, including regulation of actin cytoskeleton, focal adhesion, and ECM–receptor interaction, indicating a potential involvement of structural and signaling components in the infectious context observed. The appearance of the “Coronavirus disease—COVID-19” pathway should be interpreted with caution, as it reflects the presence of proteins commonly involved in broader inflammatory and immune processes rather than a direct association with viral infection.

Other identified pathways were associated with endocrine, infectious, and inflammatory responses, such as thyroid hormone synthesis, *Staphylococcus aureus* infection, AGE–RAGE signaling pathway in diabetic complications, and amoebiasis. Although represented by fewer proteins, these findings reinforce the functional versatility of the identified serum proteome and suggest possible crosstalk between immune-inflammatory and metabolic pathways in *Didelphis aurita*. Taken together, these enrichment results indicate that both protease regulation and vascular–inflammatory processes are central to the systemic response of *D. aurita* to *Hepatozoon* infection, consistent with the functional classifications described above, and supporting its relevance as a sentinel species in ecoimmunological studies.

## 4. Discussion

This study aimed to characterize the serum proteomic profile of *Didelphis aurita*, with a focus on the systemic response to natural infection by *Hepatozoon* sp., using mass spectrometry and functional protein annotation approaches. To the best of our knowledge, this is the first report describing the serum proteomic profile of opossums naturally infected with hemoparasites of the genus *Hepatozoon*, adding novelty and relevance to the investigation. Considering that *Didelphis aurita* frequently inhabits peri-urban and human–wildlife interface areas, understanding the systemic response of this species to parasitic agents with potential zoonotic impact contributes not only to marsupial ecoimmunology but also to public health surveillance [28].

The proteomic analysis revealed a diverse set of proteins associated with key processes such as vascular homeostasis, inflammatory response, proteolytic regulation, and oxidative stress resistance. The identification of 67 proteins with distinct distribution between *Hepatozoon*-positive and -negative groups indicates that infection by this hemoparasite significantly impacts the circulating proteomic profile of the species.

Among the identified proteins, those with the highest relative abundance were albumin, hemoglobin alpha and beta-M subunits, and the venom inhibitors DM43 and DM64. The presence of these proteins, as expected in serum, reflects their central roles in transport, redox balance, and protease regulation, as well as a possible adaptive response to parasitic infection [29,30].

The complement and coagulation cascades pathway, highly enriched in the KEGG analysis, included several key proteins involved in hemostasis, such as FGA, F5, PLG, and SERPIND1. The activation of these pathways may be directly related to *Hepatozoon* infection, since this parasite develops within erythrocytes and can promote endothelial alteration, tissue damage, and systemic inflammatory responses, which often lead to coagulation and complement activation in vertebrate hosts [31,32].

The FGA, for instance, is the precursor of fibrin and the final substrate in the coagulation cascade, playing a critical role in clot formation and vascular repair (Vilar et al., 2020) [33]. The F5 acts as a cofactor in the prothrombinase complex, catalyzing the conversion of prothrombin into thrombin, an essential step for fibrin generation and platelet activation in inflammatory contexts [34]. Plasminogen, the precursor of plasmin, contributes to fibrinolysis and regulates thrombus resolution, supporting tissue remodeling and the control of inflammation in chronic infections [35].

In this context, the abundance of these proteins may reflect a compensatory activation of coagulation, commonly observed in parasitic infections involving erythrocyte rupture, cytokine release, and endothelial interactions [36,37]. The balance between coagulation and fibrinolysis observed in the serum of *Didelphis aurita* may represent a host strategy to preserve vascular homeostasis during chronic infection [38].

The SERPIND, a potent inhibitor of serine proteases such as thrombin and factor Xa, serves as a critical negative regulator of coagulation [39]. Its high levels in serum may reflect a host response aimed at limiting thrombotic activation triggered by parasite-induced inflammation or endothelial damage [40]. This mechanism is reinforced by the presence of other serine protease inhibitors (SERPINs), such as SERPINA1, SERPINA7, and SERPINF2, which also regulate proteolytic cascades involved in both coagulation and inflammation [41,42]. The coordinated action of these inhibitors highlights the physiological effort to maintain proteolytic balance and prevent tissue damage [43,44].

Comparative proteomic investigations in other protozoan infections provide a useful framework for interpreting our findings. In *Plasmodium* spp., serum proteomics has revealed host responses involving acute-phase proteins, complement activation, and oxidative stress mediators, which parallel the enrichment of coagulation and redox-related proteins observed here [45]. Similarly, in *Trypanosoma cruzi* infection, proteomic studies have identified alterations in extracellular matrix proteins, immune modulators, and antioxidant enzymes, reflecting tissue remodeling and chronic inflammation [46,47]. In *Leishmania* spp. infections, host proteome profiling has highlighted changes in complement components and protease inhibitors, consistent with parasite evasion strategies and persistent immune activation [48]. These parallels reinforce the notion that hemoparasitic infections, despite phylogenetic differences among pathogens, converge on shared systemic pathways such as coagulation, protease regulation, and oxidative balance. However, the unique detection of venom inhibitors (DM43/DM64) in *D. aurita* underscores a potentially marsupial-specific adaptation, suggesting that while general host responses may be conserved, lineage-specific mechanisms can emerge in distinct evolutionary contexts [49].

Beyond coagulation, other significantly enriched processes such as wound healing, hemostasis, regulation of blood coagulation, and the platelet alpha granule lumen also involved several of the identified proteins, suggesting their coordinated roles in tissue injury response, systemic inflammation, and extracellular matrix remodeling [50]. These processes are particularly relevant in chronic hemoparasitic infections, where persistent cellular damage and repair mechanisms are activated [51].

The presence of VTN, FN1, and APOH reinforces the involvement of these components in cell adhesion, inflammatory signaling, and endothelial stability. Vitronectin regulates fibrinolysis and integrin-mediated adhesion, facilitating cell migration and repair [52]. Fibronectin contributes structurally to the extracellular matrix and mediates interactions between cells and plasma proteins [53,54]. APOH, also known as beta-2-glycoprotein I, participates in endotoxin neutralization, complement regulation, and recognition of oxidized phospholipids, playing a role in immune surveillance and inflammatory modulation [55].

Together, these enriched pathways and associated proteins suggest that the serum proteomic profile of *Didelphis aurita* reflects not only a hemostatic response, but also an integrated response involving immune control and tissue repair, potentially driven by the chronic presence of the parasite and its physiological consequences within erythrocytes.

Additionally, the detection of proteins with peptidase regulatory and antioxidant activities, such as GPx and HBB, points to compensatory mechanisms activated in response to oxidative stress and inflammation during infection [56,57,58]. The predominant localization of these proteins in extracellular vesicles, secretory granules, and blood microparticles further suggests intercellular signaling roles and systemic coordination of defense responses [59,60]. In marsupials, this reliance on extracellular vesicle–mediated signaling may represent a distinctive immune strategy, differing from placental mammals that more heavily depend on cytokine-driven systemic modulation [61,62]. Such a mechanism could reflect evolutionary adaptations to the unique ecoimmunological pressures faced by neotropical opossums, and it warrants targeted investigation in future studies.

Despite the novelty of these findings, some limitations must be acknowledged. The use of pooled serum samples ensured sufficient protein yield and reduced technical variability, but it precluded assessment of inter-individual differences and limited statistical comparisons. The relatively small sample size (9 positive and 10 negative animals) also constrains the generalizability of the results. Furthermore, the qualitative nature of the analysis—based on presence/absence rather than quantitative abundance—restricts the interpretation of exclusive proteins to exploratory markers. Finally, the lack of orthogonal validation techniques (Western blot, ELISA) reduces the confirmatory strength of our conclusions. These limitations do not undermine the descriptive value of the dataset, but they highlight the need for cautious interpretation.

Based on the proteins identified, several candidates emerge as potential biomarkers. SERPINs (SERPIND1, SERPINA1) and fibrinogen fragments may serve as indicators of chronic inflammation and thrombotic risk during *Hepatozoon* infection [33,39,40]. The venom inhibitors DM43 and DM64, unusually abundant in *D. aurita*, represent intriguing candidates for marsupial-specific adaptations and could be explored as markers of ecoimmunological resilience [4,49]. Proteins such as FN1, VTN, and APOH, linked to extracellular matrix stability and endothelial function, may provide insights into tissue remodeling and host vascular responses [47,50,54,55]. Future studies should test these proteins as diagnostic markers, tools for ecoimmunological surveillance, or targets for comparative immunology in wildlife health monitoring.

Altogether, our findings indicate that *Didelphis aurita* serum contains a functionally robust proteomic profile, enriched in proteins involved in coagulation regulation, immune modulation, and oxidative defense. The coexistence of pro-coagulant, fibrinolytic, and inhibitory mediators reveals a dynamic regulatory network likely essential to mitigating hemoparasite-induced damage. Importantly, beyond their descriptive novelty, the identified proteins hold potential utility as biomarkers and ecoimmunological indicators, expanding the applicability of proteomics to marsupial biology, wildlife health surveillance, and zoonotic risk assessment.

## 5. Conclusions

This study presented, for the first time, the characterization of the serum proteomic profile of *Didelphis aurita* naturally infected with *Hepatozoon* sp., revealing proteins with central roles in blood coagulation, inflammatory response, protease regulation, and antioxidant defense. Functional analyses indicated the coordinated activation of pathways associated with hemostasis, wound healing, and immune modulation, likely related to the systemic effects of intraerythrocytic infection.

Importantly, beyond descriptive novelty, the dataset highlights potential biomarkers of infection and host adaptation. SERPINs (SERPIND1, SERPINA1) and fibrinogen fragments may serve as indicators of chronic inflammation and thrombotic risk, while plasminogen and vitronectin point to compensatory mechanisms balancing coagulation and tissue repair. The high abundance of venom inhibitors DM43 and DM64, unusual in serum proteomes, suggests a marsupial-specific adaptation that could be explored as a marker of ecoimmunological resilience. Together, these proteins illustrate the interplay of conserved host responses and lineage-specific mechanisms during hemoparasitism. Although protein validation through complementary methods was not performed, and the pooling strategy limited inter-individual comparisons, the results provide a robust exploratory overview that expands the understanding of marsupial immunophysiology. These findings establish a foundation for future biomarker validation, targeted proteomic approaches, and applications in wildlife health monitoring and zoonotic risk assessment, particularly in species occupying peri-urban and human–wildlife interface environments.

## Figures and Tables

**Table 1:** Serum proteins identified by LC-MS/MS in *Didelphis aurita*, with accession numbers, molecular weight (MW), relative abundance (%), and group distribution: P (positive for *Hepatozoon* sp.), N (negative).

N°	Protein ID	Protein	MW (kDa)	Relative Abundance (%)	Group
1	A0A0B4J1D9	Ig-like domain-containing protein	12.1	2.0	P, N
2	A0A1L2DW88	C3-beta-c	184.4	4.0	P, N
3	A0A5F8G1Y1	Histone H2B	13.9	0.0	N
4	A0A5F8G575	Fibronectin	263.6	1.0	P
5	A0A5F8G606	Glutathione peroxidase	26.4	2.0	P, N
6	XP_001364584	Predicted: serotransferrin	80.2	3.0	P, N
7	A0A5F8GEV2	Transgelin	22.3	0.0	N
8	XP_007487476	PREDICTED: complement component C7	88.1	1.0	P
9	XP_007496052	PREDICTED: complement factor I isoform X1	66.0	1.0	P
10	XP_001373317	PREDICTED: fetuin-B	41.7	0.0	P
11	A0A5F8GU72	Serpin family F member 2	51.7	1.0	P
12	A0A5F8GVK6	C4a anaphylatoxin	198.8	4.0	P, N
13	A0A5F8GVX1	Carboxypeptidase B2	47.7	0.0	P
14	A0A5F8H055	Thioredoxin domain-containing protein	22.0	0.0	N
15	A0A5F8H3J0	Ig-like domain-containing protein	21.6	1.0	N
16	XP_007500536	PREDICTED: inter-alpha-trypsin inhibitor heavy chain H3	93.1	1.0	P
17	A0A5F8H662	Gc-globulin	56.2	0.0	P
18	XP_001380249	PREDICTED: inter-alpha-trypsin inhibitor heavy chain H4-like isoform X2	71.0	1.0	P
19	A0A5F8H890	SERPIN domain-containing protein	47.8	0.0	P
20	A0A5F8H8I8	Inter-alpha-trypsin inhibitor heavy chain 1	96.4	0.0	P
21	A0A5F8HA42	Apolipoprotein M	22.1	2.0	N
22	XP_007491633	PREDICTED: apolipoprotein E	33.5	1.0	P, N
23	A0A5F8HGF5	Actin-depolymerizing factor	81.4	0.0	N
24	A0A5F8HGH4	Antithrombin-III	56.8	2.0	P
25	XP_001380867	PREDICTED: apolipoprotein A-I	30.2	1.0	N
26	F6QPV8	Apolipoprotein H	38.6	0.0	P
27	F6QTH2	SMB domain-containing protein	53.6	1.0	P
28	F6R154	C-type lectin domain-containing protein	22.8	1.0	N
29	XP_001371162	PREDICTED: ceruloplasmin isoform X2	120.2	2.0	P
30	XP_001380874	PREDICTED: apolipoprotein A-IV	42.5	0.0	P
31	F6UL60	Vitamin K-dependent protein	76.1	0.0	P
32	XP_001369603	PREDICTED: complement C5	185.3	2.0	P
33	F6UX07	Clusterin	56.2	1.0	P
34	F6W869	Coagulation factor V	243.8	0.0	P
35	F6X165	ADF-H domain-containing protein	16.0	0.0	N
36	XP_001375856	PREDICTED: proteoglycan 4 isoform X1	142.4	0.0	N
37	F6YK01	Serpin family D member 1	70.6	1.0	P
38	F6Z008	Angiotensin 1-10	52.6	0.0	P
39	F7A2F0	Lysozyme	16.9	0.0	N
40	F7B2I8	Receptor protein-tyrosine kinase	135.3	0.0	P
41	XP_007481728	PREDICTED: LOW QUALITY PROTEIN: C-reactive protein	10.3	0.0	N
42	XP_007503682	PREDICTED: alpha-2-macroglobulin isoform X1	165.6	3.0	P
43	XP_001371538	PREDICTED: complement component C6 isoform X1	105.4	0.0	P
44	XP_001364858	PREDICTED: serum albumin	68.1	13.0	P, N
45	F7BVM7	Haptoglobin	45.9	1.0	P, N
46	XP_001370529	PREDICTED: complement C1s subcomponent	77.6	0.0	P
47	XP_001365240	PREDICTED: inter-alpha-trypsin inhibitor heavy chain H2 isoform X1	107.5	0.0	P
48	F7CJ60	Fibrinogen alpha chain	91.2	1.0	P, N
49	F7DMM7	Profilin	15.0	1.0	N
50	F7DSP2	Coagulation factor X	58.0	0.0	P
51	F7EI80	CN hydrolase domain-containing protein	56.2	0.0	P
52	F7EVI4	NAD(P)-bd_dom domain-containing protein	22.0	0.0	P, N
53	F7F243	Plasminogen	91.2	2.0	P
54	F7F3D5	C3/C5 convertase	159.7	1.0	P
55	F7FD92	Hemopexin	50.3	1.0	P
56	F7FPF7	Serum amyloid A protein	14.3	1.0	N
57	F7FZ45	Peptidyl-prolyl cis-trans isomerase	17.9	0.0	N
58	F7G7P1	Complement component C9	70.4	1.0	P
59	K7E0U3	Retinol-binding protein	23.9	0.0	N
60	P01976	Hemoglobin subunit alpha	15.3	8.0	P, N
61	P02109	Hemoglobin subunit beta-M	16.2	10.0	P, N
62	P11025	Hemoglobin subunit epsilon-M	16.3	1.0	N
63	P49141	Transthyretin	16.4	1.0	P, N
64	P82957	Venom metalloproteinase inhibitor DM43	32.4	7.0	P, N
65	Q03044	Alpha-1-antiproteinase	46.4	2.0	P, N
66	Q8HYX5	Venom metalloproteinase inhibitor DM43b	34.6	4.0	P, N
67	Q8MIS3	Venom myotoxin inhibitor DM64	56.0	7.0	P, N

## Data Availability

The data presented in this study are available from the corresponding author upon request.

## Supplementary Materials

The following are available online at https://www.mdpi.com/article/10.3390/pathogens14101042/s1, Figure S1: Giemsa-stained blood smear from a *Didelphis aurita* specimen showing intracellular *Hepatozoon* sp. gamonts (black arrows) within erythrocytes. Observed under light microscopy at 1000× magnification. Figure S2: SDS-PAGE profile of serum proteins from *Didelphis aurita* used for proteomic analysis. A 24 cm polyacrylamide gel at 10% concentration was run under denaturing conditions (Laemmli system) to assess the quality of protein extraction and quantification. Each lane corresponds to an individual sample from Hepatozoon-positive (Hep+) or Hepatozoon-negative (Hep–) animals. MW: molecular weight marker. The gel shows consistent banding patterns across samples, confirming suitability for subsequent LC-MS/MS analysis.

## Abbreviations

The following abbreviations are used in this manuscript:

SDS-PAGE	Sodium dodecyl sulfate–polyacrylamide gel electrophoresis
SISBIO	Brazilian Biodiversity Information and Authorization System
IBAMA	Brazilian Institute of Environment and Renewable Natural Resources
DTT	Dithiothreitol
BSA	Bovine serum albumin
LC-MS/MS	Liquid chromatography-tandem mass spectrometry
FDR	False discovery rate
DAVID	Database for Annotation, Visualization and Integrated Discovery
GO	Gene Ontology
KEGG	Kyoto Encyclopedia of Genes and Genomes
