# Supplementary figures and images for: Serum Proteomics Reveals Systemic Responses in Didelphis aurita Naturally Infected with Hepatozoon sp
Source: Pathogens. 2025 Oct 14;14(10):1042. doi: 10.3390/pathogens14101042 (PMC12567376; doi:10.3390/pathogens14101042)

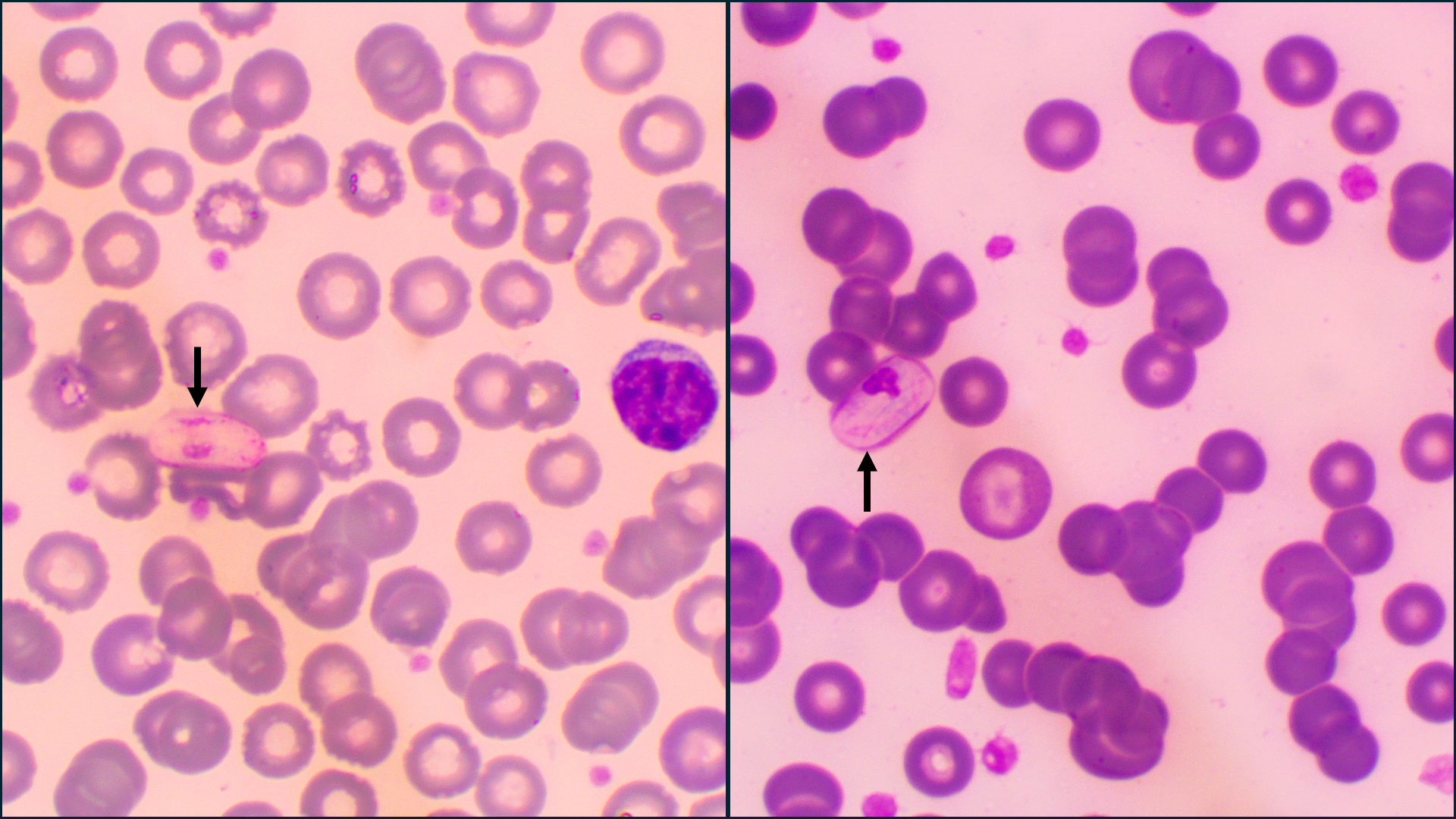

Supplement: Supplementary file 1 [file pathogens-14-01042-s001.zip › Figure S1.tiff]

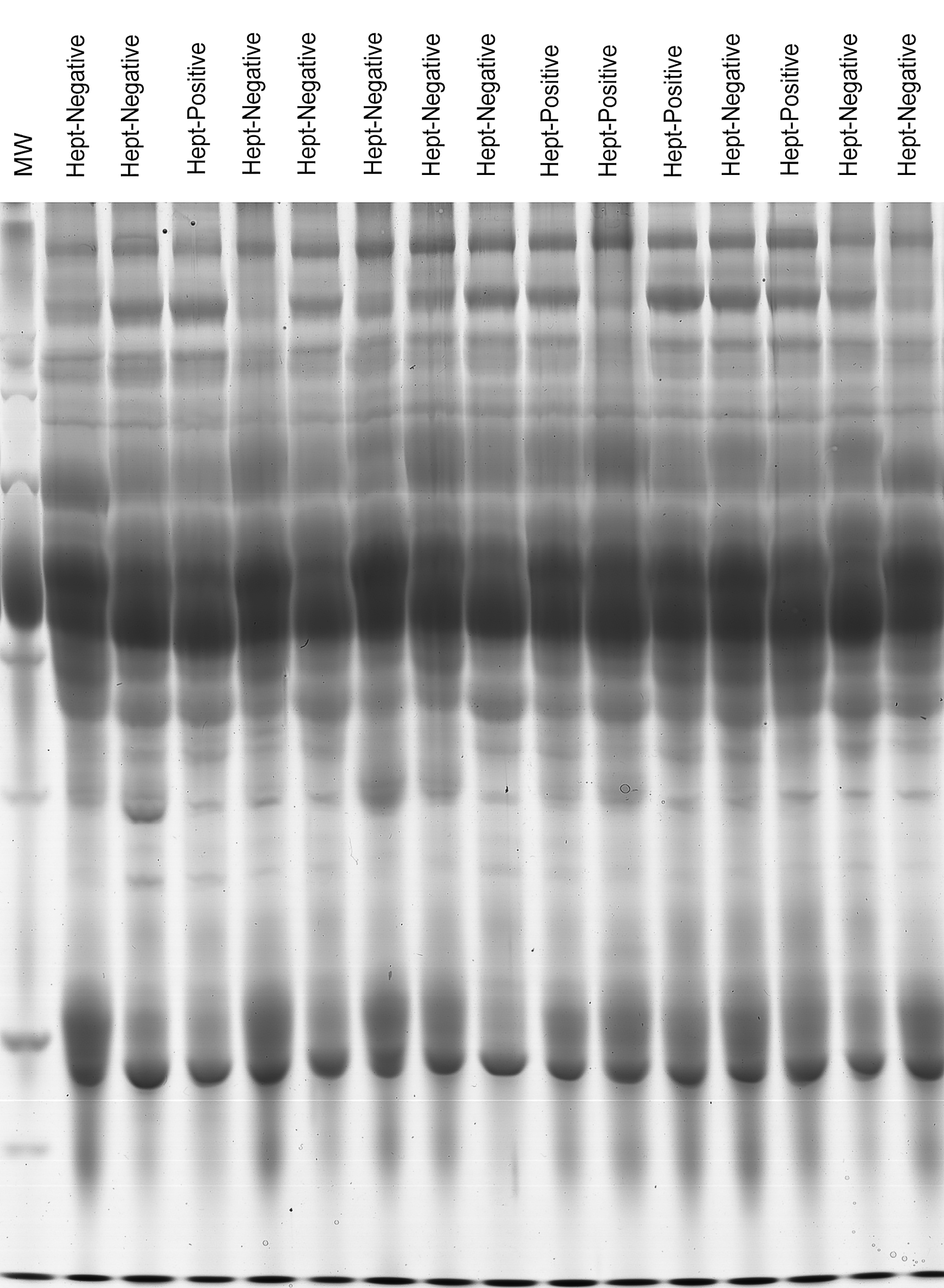

Supplement: Supplementary file 1 [file pathogens-14-01042-s001.zip › Figure S2.jpg]
